# A conceptual framework for prognostic research

**DOI:** 10.1186/s12874-020-01050-7

**Published:** 2020-06-29

**Authors:** Peter Kent, Carol Cancelliere, Eleanor Boyle, J. David Cassidy, Alice Kongsted

**Affiliations:** 1 School of Physiotherapy and Exercise Science, Curtin University, Kent St, Bentley, Perth, WA 6102 Australia; 2 Department of Sports Science and Clinical Biomechanics, University of Southern Denmark, Odense, Denmark; 3 Faculty of Health Sciences, Ontario Tech University, Oshawa, Ontario Canada; 4 Centre for Disability Prevention and Rehabilitation, Ontario Tech University and the Canadian Memorial Chiropractic College, Toronto, Ontario Canada; 5 Division of Epidemiology, Dalla Lana School of Public Health, University of Toronto, Toronto, Canada; 6 Nordic Institute of Chiropractic and Clinical Biomechanics, Odense, Denmark

**Keywords:** Prognosis, Association, Prediction, Causality

## Abstract

**Background:**

Prognostic research has many important purposes, including (i) describing the natural history and clinical course of health conditions, (ii) investigating variables associated with health outcomes of interest, (iii) estimating an individual’s probability of developing different outcomes, (iv) investigating the clinical application of prediction models, and (v) investigating determinants of recovery that can inform the development of interventions to improve patient outcomes. But much prognostic research has been poorly conducted and interpreted, indicating that a number of conceptual areas are often misunderstood. Recent initiatives to improve this include the Prognosis Research Strategy (PROGRESS) and the Transparent Reporting of a multivariable prediction model for Individual Prognosis or Diagnosis (TRIPOD) Statement. In this paper, we aim to show how different categories of prognostic research relate to each other, to differentiate exploratory and confirmatory studies, discuss moderators and mediators, and to show how important it is to understand study designs and the differences between prediction and causation.

**Main text:**

We propose that there are four main objectives of prognostic studies – description, association, prediction and causation. By causation, we mean the effect of prediction and decision rules on outcomes as determined by intervention studies and the investigation of whether a prognostic factor is a determinant of outcome (on the causal pathway). These either fall under the umbrella of exploratory (description, association, and prediction model development) or confirmatory (prediction model external validation and investigation of causation). Including considerations of causation within a prognostic framework provides a more comprehensive roadmap of how different types of studies conceptually relate to each other, and better clarity about appropriate model performance measures and the inferences that can be drawn from different types of prognostic studies. We also propose definitions of ‘candidate prognostic factors’, ‘prognostic factors’, ‘prognostic determinants (causal)’ and ‘prognostic markers (non-causal)’. Furthermore, we address common conceptual misunderstandings related to study design, analysis, and interpretation of multivariable models from the perspectives of association, prediction and causation.

**Conclusion:**

This paper uses a framework to clarify some concepts in prognostic research that remain poorly understood and implemented, to stimulate discussion about how prognostic studies can be strengthened and appropriately interpreted.

## Background

Questions of prognosis are among the most important for patient care [1]. Prognostic research serves many purposes. It aims to describe the natural history and clinical course of health conditions, and it provides evidence about the burden of disease. It is also a method to investigate variables associated with health outcomes of interest. Prognostic research also can establish an evidence-based understanding of an individual’s probability of developing different outcomes and can inform the development of interventions and policies to improve the diagnosis of health conditions and management of patients [1, 2]. It can also provide indications about which prognostic variables appear to be on the causal pathway of a health condition or outcome [3]. Prognostic research spans different areas of inquiry from classical epidemiology and public health through to clinical practice and stratified care, each with its particular focus but also with considerable overlap of shared methods.

In our areas of expertise in neck and back pain, traffic injuries, and mild traumatic brain injury, there are currently few examples of the implementation of prognostic research resulting in improved patient care [4–8], and critical appraisal of prognostic studies in these areas has clearly demonstrated the need to improve the conduct, design, analysis and interpretation of prognosis research [4, 5, 9–12]. For example, in a large international systematic review published in 2004 to determine the prognosis after mild traumatic brain injury [5], only 28% of the studies were of sufficiently high quality (i.e., low risk of bias) to be included in a best-evidence synthesis. A decade later, and despite calls to improve the methodological quality of prognostic research, the acceptance rate by the international systematic review group who updated these findings, remained similarly low at 34% [4]. Recent systematic reviews regarding prognosis in whiplash [13], cancer [14, 15] and cardiovascular disease [16, 17] also report methodological problems in many studies. Clearly, we need to do better because poorly conceived and reported research is wasteful, potentially misleading and arguably not ethical.

To address this, there has been a significant effort to improve the design, conduct and reporting of prognostic studies [1, 9, 18–34]. In 2013, the Prognosis Research Strategy (PROGRESS) group published a series of papers [1, 23, 31, 34], and in 2019 a comprehensive book [35], that together outline issues of importance for prognostic studies and make recommendations to improve current prognostic research standards. Also, the ‘Transparent reporting of a multivariable prediction model for individual prognosis or diagnosis (TRIPOD) statement’ from 2015 provides helpful guidance for developing, testing and reporting prediction models [30], and the CHecklist for critical Appraisal and data extraction for systematic Reviews of prediction Modelling Studies (CHARMS) [36, 37] provides guidance for evaluating prediction models.

Whereas PROGRESS, TRIPOD and CHARMS focus on descriptive epidemiology and prediction of outcome, others have emphasised the importance of differentiating between this and research aimed at establishing causal relationships [22, 33]. Understanding the differences between prediction and causal research questions is very helpful for designing, conducting and communicating clinical research.

For many years, we have been teaching a post-graduate course on prognostic methods and our experience is that there are a number of conceptual areas that students often misunderstand. We have also observed, when critically appraising and reviewing manuscripts, that these misunderstandings are also often present in studies by experienced researchers [38].

Therefore, the aim of this article is to show how different types of prognostic research are related to and inform each other, to differentiate exploratory and confirmatory studies, to clarify statistical measures and inferences appropriate at different types of prognostic research, to discuss moderators and mediators, and to show how important it is to understand the importance of study design and the differences between prediction and causation. Working examples from selected health conditions will illustrate many of these concepts.

## Main text

**Fig. 1:**
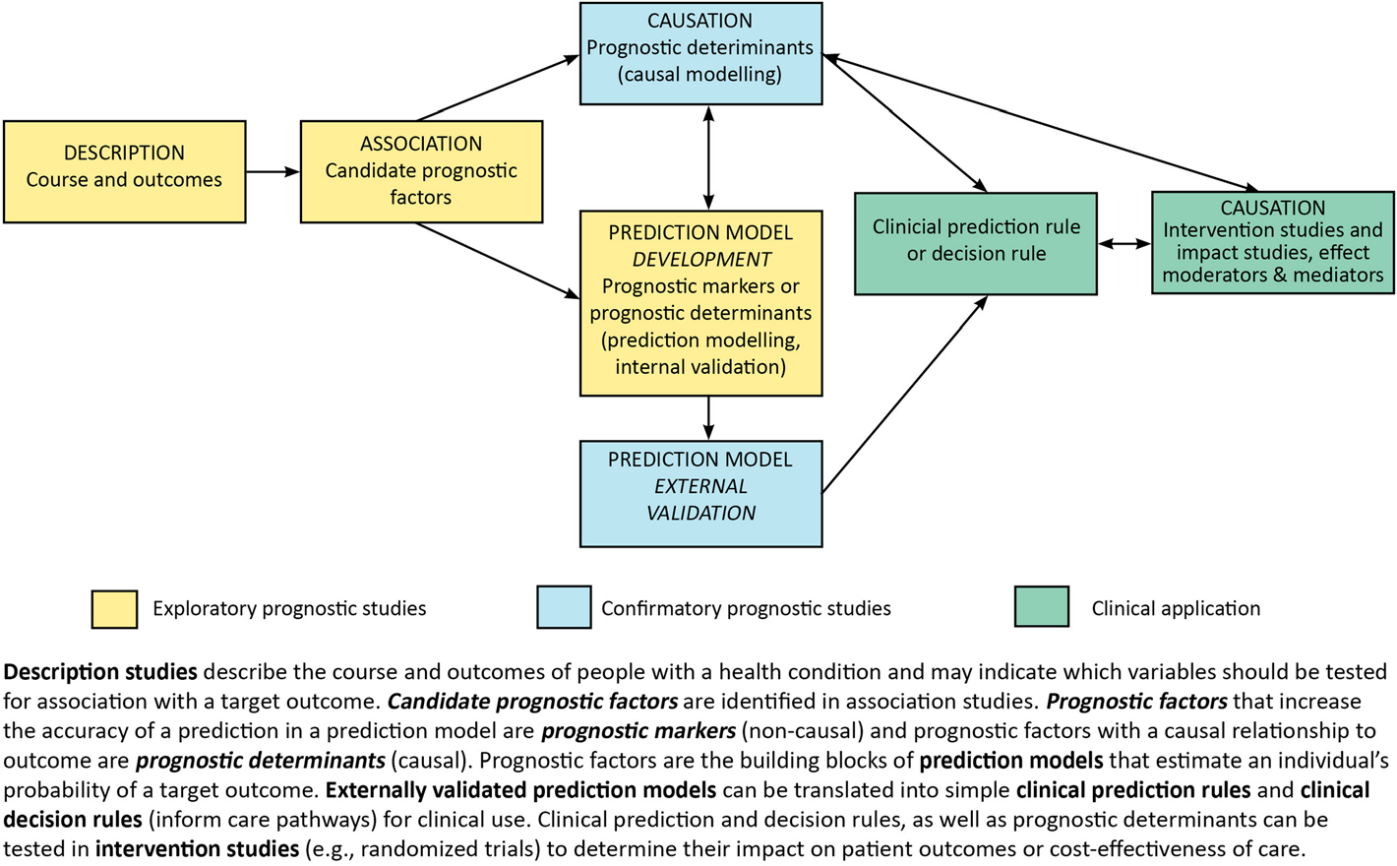
Prognostic research conceptual framework

We have found that the use of a visual conceptual framework (see Fig. 1) helps clarify the links and differences between the concepts of prognostic research and causation research.

In general, we propose there are four main objectives of prognostic studies: description, association, prediction, and causation. These objectives either fall under the umbrella of **exploratory** studies (description, association, and prediction model development) or **confirmatory** studies (prediction model external validation and investigation of causal relationships). Most prognostic studies published in our research fields have been exploratory [4, 9, 39–41]. These are initially carried out when little is known about a health condition. As indicated by the unidirectional arrows, exploratory studies are an essential first step towards undertaking a confirmatory study.

An overall summary of the types of studies discussed is contained in Table 1. The concepts detailed in the Table are explained in the following sections.

**Table 1:** Studies of prognosis

Research Purpose	Study Design	Analysis	Performance Measures	Model interpretation *(From* Fig. 2b *multivariable regression model)*	Application
**Exploratory prognostic studies**					
**2.1.1. Description**					
To describe the outcomes and course of people with a health condition. E.g., What is the course of recovery for adults with acute back pain (within 7 days of onset)?	Cohort (ideally an inception cohort^a^)	Descriptive statistics. For example, measure pain severity and function at pre-specified time intervals. Trajectory analysis can also be useful.	N/A	N/A	Understanding the course of a disease or exploring trajectories of recovery. May also indicate which outcomes could be tested for an association with candidate prognostic factors
**2.1.2 Association**					
To identify candidate prognostic factors (prognostic markers /determinants). E.g., What factors are associated with disability in adults 12 months after onset of an episode of back pain?	Cohort (ideally an inception cohort^a^) and case-control studies.	Ideally, a multivariable model focusing on the strength of association between each candidate prognostic factor and an outcome.	Strength of association: the size of the beta-coefficient, odds / risk / hazard ratio, the width of the 95% confidence interval, and the statistical significance for each candidate prognostic factor	All three factors are associated with disability at 12 months: back pain duration (13.2, 95%CI 11.0, 15.5), baseline disability (0.29, 95%CI 0.25, .33), recovery expectations (− 3.2, 95%CI − 3.5, − 2.8). *Note: the strength of association depends on other factors in model and are not directly comparable when prognostic factors are measured on different scales*	Indicate which prognostic factors might be considered for use in predictive models and causal research
**2.1.3 Prediction Model Development**					
To determine predictors (prognostic markers/determinants) of an outcome. What is the probability of an outcome? E.g., What predicts disability in adults 12 months after onset of an episode of back pain?	Inception cohort^a^, although sometimes a prevalence cohort is used if the intended clinical application of the model requires it	Multivariable model	Collective predictive ability of a set of predictors. Common measures of predictive ability include discrimination, calibration, R^2^.	Prediction model with the 3 predictors (back pain duration, baseline disability, and baseline recovery expectations) predicts disability at 12 months (adjusted R^2^ = 0.39)	Identification of chosen model is followed by the need for testing its external validity
**Confirmatory prognostic studies**					
**2.2.1 Prediction Model External Validation**					
To determine if the prediction model predicts well in external populations. E.g., What predicts disability in adults 12 months after onset of an episode of back pain?	Cohort (as above)	Apply coefficients for each predictor (from model development) to this new cohort	Model performs well in this independent cohort (similar to how it performed in development cohort). Common measures of model performance include model fit, discrimination, calibration and shrinkage.	N/A	Translate into clinical prediction/decision rules
**2.2.3 Studies of causation**					
To determine if a candidate prognostic variable is a prognostic determinant (cause) of an outcome. E.g., Is recovery expectation a prognostic factor of disability 12 months after onset of an episode of back pain?	Inception cohort^a^	Test pre-specified hypothesis. Multivariable model. There are many research designs for different causal questions. One simple design is to determine whether an independent association exists between the potential prognostic determinant and an outcome, while controlling for potential confounders	Strength of association (effect estimate), its 95% confidence interval, and p-value in the presence of potential confounders	Recovery expectation is a prognostic determinant (cause) of disability at 12 months (−3.18, 95% CI −3.5, −2.8) independent of back pain duration and baseline disability.	Develop and test interventions targeted at the modifiable prognostic determinant. For example, to test whether improving patients’ expectations results in better outcomes
**Clinical application**					
**2.2.2 Clinical Prediction or Decision Rules**					
Clinical prediction rule: A version of the prediction model that has been simplified for clinical use. A tool used in the clinic that helps inform patients and clinicians about the probability of an outcome. Clinical decision rule: assists clinicians with decision-making and care pathways. E.g., A prediction rule indicating which people have a higher probability of responding well to a particular therapeutic intervention.	A before and after design		Feasibility, clinician and patient acceptance, estimates of likely effect on patient outcomes and/or health system outcomes		Determine whether effect should be subsequently tested in an intervention study.
To determine the impact of using a clinical prediction/decision rule on patient outcomes or cost-effectiveness of care. E.g., What is the impact of implementing the use of a clinical decision rule in adults with back pain?	Randomised controlled trial		Measures of impact: clinician adoption rates, clinician and patient acceptability, change in decision-making, improvement in patient, health system and economic outcomes		Recommend clinical prediction/decision rules for use in clinical practice.

^a^Inception cohort: participants are incepted at a uniform time (zero time), such as at the onset of a condition of interest or new episode of a condition of interest or onset of care-seeking, and are then followed over time for the development of outcome(s)

**Fig. 2:**
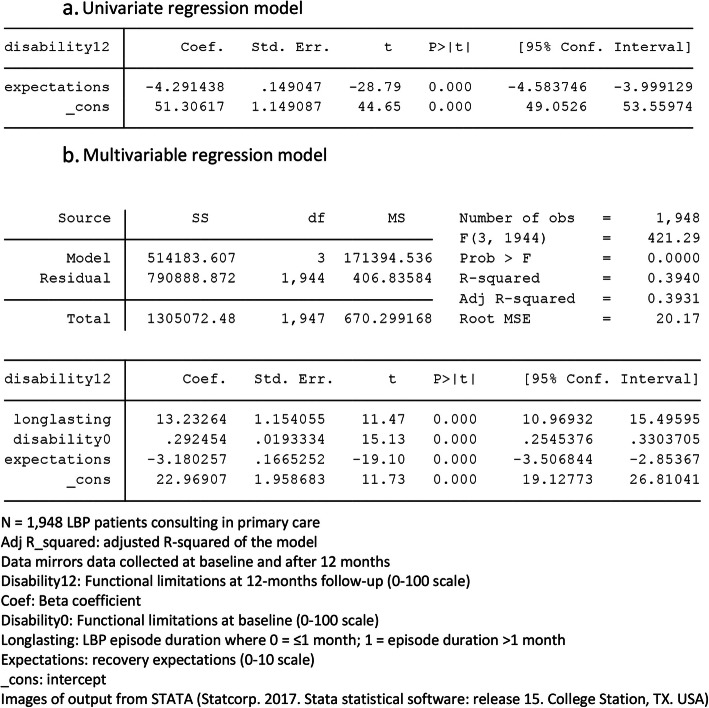
Example univariate and multivariable regression models

Statistical models in prognostic research often involve the use of simple univariate and more complex multivariable regression models. Our experience is that there frequently are conceptual misunderstandings about (i) what can be inferred from a multivariable model from the perspectives of association, prediction and causation, and (ii) what statistical measures in a multivariable model are meaningful from the perspectives of association, prediction and causation. The central misunderstandings here are a lack of recognition that (i) it is the type of research question, not the statistical model, that drives the interpretation, and (ii) the type of research question determines where you are on our framework and the statistical measures that are relevant. As examples to illustrate this later in the paper, Fig. 2a and b show the output from simple univariate and multivariable linear regression models of artificial data from 1948 people with back pain. They have an outcome or dependent variable (functional disability at 12 months follow-up) and up to three independent variables (duration of back pain, functional disability at baseline, and recovery expectations at baseline). We will refer back to these models in the subsequent sections about how different research questions markedly influence interpretation of such models.

### Exploratory prognostic studies

**Description** studies describe the course or outcomes of people with a particular health condition, including dichotomous descriptors such as the proportion of people who acquire the health condition, who recover, or who develop a long-term consequence. In the PROGRESS series, these were referred to as ‘Type 1: fundamental prognosis research’. For a visual representation of the overlap between the phases of research proposed in the PROGRESS series and those in our conceptual framework, see Additional file 1.

*For instance, in back pain a descriptive study might describe the development of pain intensity from onset until 12 months later, focusing on the population average or individual trajectories.*

#### Useful statistics in descriptive prognostic studies

Simple descriptive statistics (means, medians, proportions) and measures of variability (standard deviations, inter-quartile ranges, 95% confidence intervals)) are common in descriptive studies. Also useful are descriptions of trajectories, such as time-series, survival curves analysis and latent growth analysis.

Studies of **association** identify associations between variables and outcomes of interest. They are required when it is unclear which variables are potentially important in predicting an outcome for people in a specific population or when causal components of an outcome are not fully known. Association studies identify *candidate prognostic factors* which would be further tested in prediction or causation studies. These studies are included in what the PROGRESS series referred to as ‘Type 2: prognostic factor research’.

*For instance, an association study might demonstrate an association between baseline recovery expectations and improvements in pain over the follow up period. Subsequent studies might investigate the predictive value of expectations in identifying those that recover (a prediction study) or if expectations are on the causal pathway of recovery (a causation study).*

Many studies of association have used data collected at a single time point (cross-sectional data) where the notional outcome is collected at the same time point as the candidate prognostic factor(s) of interest. These types of studies can only be used to set *very* tentative hypotheses about potential associations between candidate prognostic factors and outcomes. A much stronger and preferable design is to use cohort data where participants initially do not have the outcome and outcome is measured at a later time point than the candidate prognostic factors. In an inception cohort study, participants are incepted at a uniform time (zero time), such as at the onset of a condition of interest, onset of an episode of a condition of interest, or onset of care-seeking, and are then followed over time for the development of the outcome. This ensures that the outcome occurs after the assessment of the candidate prognostic factor and that mild and severe cases are included in the study preventing prevalence-incidence bias. Candidate prognostic factors can also be identified from case-control studies where cases, who have an outcome, are retrospectively compared to controls, who do not have the outcome, using data about candidate prognostic factors that were collected at some earlier timepoint.

#### Useful statistics in studies of association

**Fig. 3:**
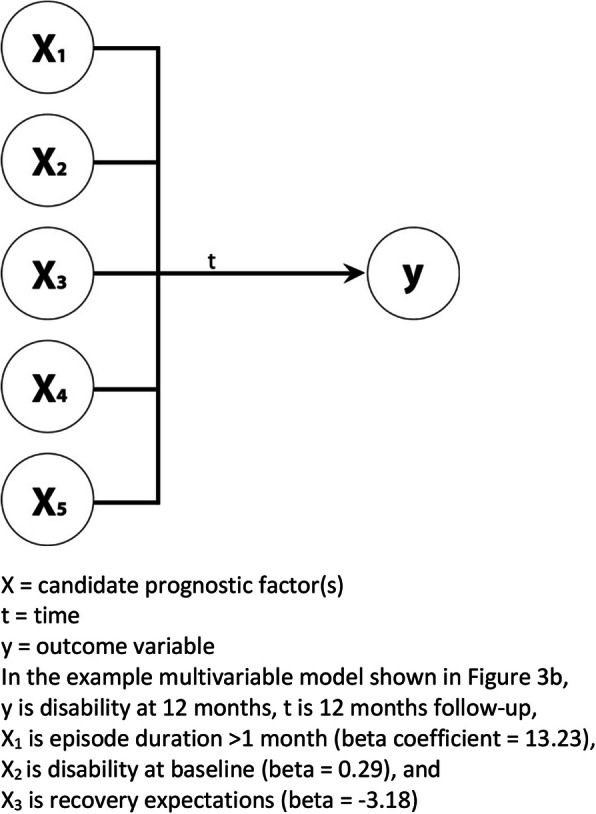
Concept of multivariable model of association

Figure 3 shows the basic concept of models of association. It quantifies the relationship between one or more candidate prognostic factors (x) collected at one time point with an outcome of interest (y) at some later time point.

The simplest form of a study of association is determining the univariate relationship between a single candidate prognostic factor and an outcome. In that circumstance, the three pieces of information that are meaningful are the strength of the association (size of the coefficient, odds ratio or risk ratio), its confidence interval (certainty of the estimate) and the *p*-value of the candidate prognostic factor. In the example regression model in Fig. 2a, the coefficient of expectations is − 4.29, which means that people who scored 4 on the 0–10 expectation scale would on average have a functional disability change score that is 17.16 less (− 4.29 × 4) at 12 months than people who scored 0 for baseline expectations.

The screening of candidate prognostic factors by their univariate statistical significance has historically been common but this practice is now discouraged, for reasons well described by the PROGRESS group and others (such as Sun 1996) [42]. The principal reason is that, in the presence of other independent variables, a non-significant univariate association may become significant, and a significant univariate association may become non-significant, so univariate screening using *p*-values as the criterion is not dependable. For this reason, it is often relevant for studies of association to determine the simultaneous (multivariable) relationship between multiple candidate prognostic factors and an outcome, such as in Fig. 2b. In this circumstance, the three pieces of information that are meaningful remain the size of the coefficient, the confidence interval and the *p*-value for each of the candidate prognostic factors.

The interpretation of the absolute size of the p-value (rather than whether it is above a arbitrary threshold) [43] needs to take into consideration whether the available sample size was appropriate, given that its value will be smaller in large samples. Therefore, while coefficients, their confidence intervals, and *p*-values inform decisions about the strength and certainty of an association, in studies of association this should be considered to be only a screening of candidate prognostic factors and not a definitive estimate of the strength of that association.

**Prediction model development** aims to identify the best set of *predictors* of a target outcome and to predict an individual’s probability of experiencing that outcome [31, 34]. Predicting future outcomes is the cornerstone of prognostic research. In the PROGRESS series, these were referred to as ‘Type 3: prognostic model research’.

Just as in studies of association, Fig. 2b is equally applicable as the same basic concept of models of prediction, as they both quantify the relationship between one or more candidate prognostic factors (x) collected at one time point with an outcome of interest (y) at some later time point. However, in the development of prediction models, a *set* of predictors is identified which together explain the most variance in the outcome. So, in prediction studies the explained variance of the outcome of the whole prediction model becomes important, unlike studies of association where the focus is on the predictive strength of association between individual variables, even though their statistical models might be identical.

*For instance, recovery expectations would be predictive of back pain intensity if adding it to a multivariable model with other candidate variables improved the overall predictive strength of the model*

Similar to the PROGRESS group, we define a *prognostic factor* as any measure that, among people with a given health condition is associated with a subsequent clinical outcome [31]. In addition to that, we suggest differentiating between a *prognostic determinant* that is on the causal pathway and a *prognostic marker* as a variable that predicts the outcome of interest in a prediction model and is not on the causal pathway. Of note is that this distinction is inconsequential for the purpose of building a predictive model because the pragmatic purpose of building predictive models is to find useful combinations of predictors (prognostic determinants or markers) that result in sufficiently accurate estimates of an outcome of interest at the time period(s) of interest, regardless of whether those predictors are on the causal pathway or not. None-the-less in our view, the use of these terms (determinants and markers) is not just about linguistic precision, it is about sign posting the intention of a research question and study as either about prediction or causation, as this distinction is frequently confused, and/or anchoring the selection of variables in a theoretical framework so that inferences derived from studies are more defensible.

While in principle the distinction between prognostic determinants and markers is generally inconsequential for the purpose of building a prediction model, this may not be the case when designing a tool to guide decisions about content of treatment, where a preference can be for prognostic factors that are potentially modifiable and on the causal pathway [44]. Non-modifiable prognostic factors in a prediction model can also be useful in guiding treatment decisions, for example a person’s age may impact their probability of responding to a particular treatment.

Cross-sectional data is not suitable for use in studies of prediction, not even in the development phase. Studies of prediction require prospectively collected longitudinal data where the outcome is not present at enrolment.

When developing prediction models for settings with patient populations that are heterogenous (e.g. in the duration of their health condition and/or treatment history), the influence of these differences at inception should be carefully considered. It is the case that prediction rules for clinical settings need to be relevant to the case profile of clinicians and while some clinicians routinely see patients early in their clinical course, many first see patients at highly variable points in their clinical course. Nonetheless, heterogeneity of time-zero (time-zero bias) and/or treatment history require the exploration of whether these factors are moderators of the observed prognostic effect and therefore need to be integrated into the derived prognostic models and prediction rules (such as via stratification). Effect moderation is explained in more detail below.

#### Useful statistics in studies that develop prediction models

Given that the collective predictive ability of that particular set of predictors is the focus in prediction models, useful statistics are the overall performance and fit of the model. For example, the amount of explained variance in the outcome variable as quantified by the adjusted R-squared value and estimates of model error as quantified by the Root MSE (Mean Squared Error) term are of most interest in Fig. 2b. In that example, the adjusted R-squared value is 0.3931, indicating that 39% of the variance in the outcome variable is collectively explained by the model containing those three predictors. For binary outcomes, measures such as Area Under the ROC-curve (C-statistic) and positive/negative predictive values, are used to quantify model performance.

Other statistical measures that are important for prediction models, and can be calculated in a number of ways, are calibration (the agreement between predicted and observed outcomes) and discrimination (how well predictions separate people who have and do not have the outcome of interest). Prediction model development involves building and comparing multiple models with different combinations and numbers of prognostic markers and prognostic determinants, in the search for the one best model.

**Fig. 4:**
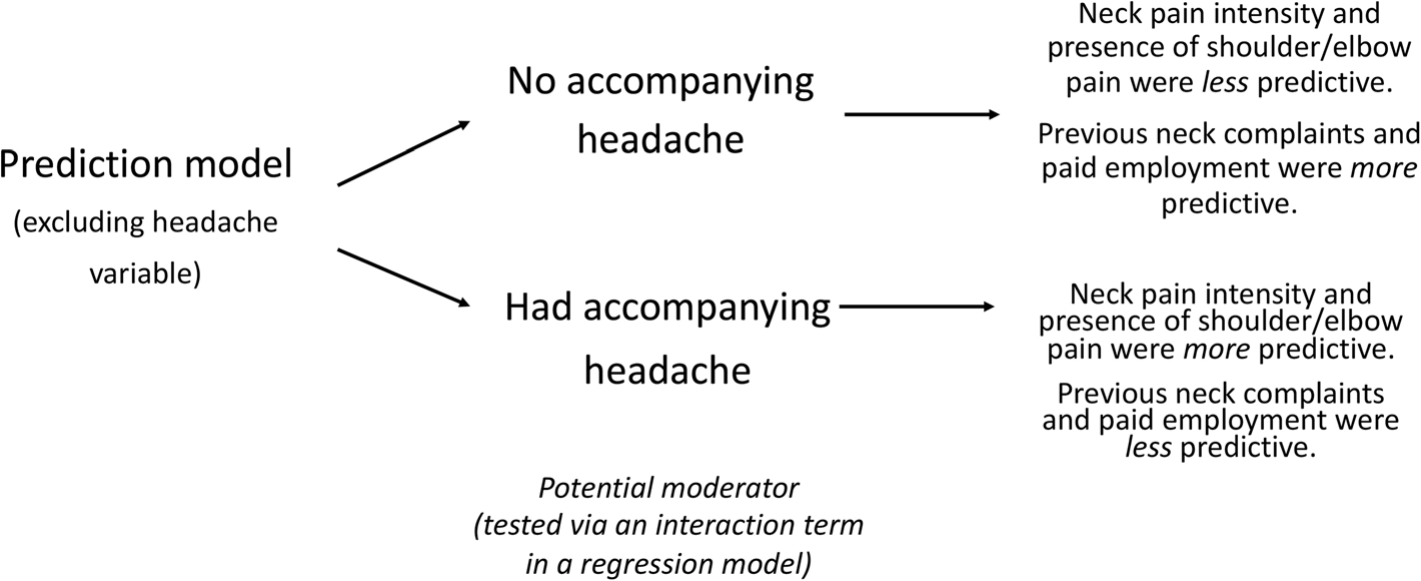
An example of a prediction model affected by a moderation variable

Effect moderation can be important in prediction studies. In this context, moderation is where the relationship between a prognostic factor and the outcome differs depending on the levels of the moderating variable. That moderation may affect one or more of the prognostic factors in a prediction model and is tested by introducing interaction terms into regression models. An example is that Schellingerhout et.al [45] found their prediction rule about the persistence of neck complaints in people following whiplash was moderated by the presence of an accompanying headache (Fig. 4).

### Confirmatory prognostic studies

Confirmatory studies include the external validation of prediction models, clinical application of prediction models (their acceptability, adoption, and effect on outcomes) and investigating whether a prognostic factor is a determinant of outcome (Fig. 1).

### External validation

After prediction models are created using one sample of individuals during model development, they need to be tested in new samples of similar individuals (i.e., external validation) [19, 39, 46].

*An external validation of a prediction model developed in one sample of patients from physiotherapy practice could be performed by applying the model in other physiotherapy clinics or other care settings seeing similar patients.*

Prediction model validation can involve using the same performance measures as used in the development phase, but now in an external sample of new people, by applying the previously derived coefficients for each predictor to the new sample. It can also involve updating and recalibrating an existing model in a new setting, which may include tweaking which predictors are in the model or their weights, and the use of additional statistical measures, such as net reclassification improvement [47]. As with prediction model development, only prospective cohort designs are relevant for model validation.

### Clinical application

The clinical application of prognostic information can include the development of **clinical prediction or decision rules**, and studies that seek to determine whether those rules do make a difference to outcomes when applied in treatment settings.

For clinical use, externally validated prediction models can be translated into simple clinical prediction rules and clinical decision rules, which inform care pathways or choice of treatment. Those rules guide the choice of treatment by providing information on the likely outcome of an individual given different interventions, whereas prognostic rules inform the likely prognosis of an individual given one treatment or care pathway. In a final stage, clinical prediction and decision rules, as well as single prognostic determinants can be tested in intervention studies (e.g. randomised clinical trials) to determine the impact of using the rule on patient outcomes and the cost-effectiveness of care or the effects of intervening on the prognostic determinant. Randomised and non-randomised impact studies can also play a role in describing the pragmatic ability of clinical rules to be adopted, change practice and improve outcomes.

*For example, stratification of back pain patients based on potentially modifiable prognostic determinants are used to guide care pathways for individual patients. The impact of such an approach is tested in randomised controlled trials or other types of implementation studies* [6, 48]*.*

### Studies of causation

Investigating prediction and causation involve different research questions and are often confused. That confusion leads to the error of making causal inferences when performing a prediction study and vice-versa. This has been well described by Hayden et.al 2008 [21], Herbert 2014 [22] and Shmueli 2010 [33]. In this context we have used the terms ‘prediction/causation’, for the same concepts Hayden et.al 2008 [21] used ‘prediction/explanatory’ and Herbert 2014 [22] used ‘prognosis/aetiology’, however the meaning is the same.

As described above, prediction studies aim at estimating an individual’s likely outcome or course of disease as precisely as possible. In that context, the potential causal relationship between prognostic factors and outcome is only of interest to the extent that some prognostic determinants can be strong predictors and therefore might be worth considering for inclusion (represented by the bidirectional arrow extending from the ‘causation modelling’ box to the ‘prediction model development’ box in Fig. 1). In contrast, in causation research, what we care about is exactly the extent to which a prognostic determinant or exposure (which may be a potential target for interventions) affects or determines outcome or course of a given health condition. That information is important for understanding determinants of recovery.

Causation studies can take various forms, from simple studies of independent associations between one prognostic factor of interest and an outcome with adequate control for confounding (used as an example below) through various types of studies of mediation, multi-causation, and effect moderation (using methods such as causal and acyclic diagrams and structural equation modelling). The central concepts are that causal studies test pre-specified hypotheses and one or more pre-specified models about causal relationships, while controlling for potential confounding factors. In contrast, there is no hypothesis-testing in studies of prediction, nor any need to control for confounding, as confounding is not a consideration in prediction.

*For example, the potential causal relationship between recovery expectations and change in back pain intensity would be investigated in models that account for confounding and perhaps explore differential effects of expectations in subgroups of patients. If a causal relationship is identified, intervention studies could test if modifying patients’ expectations leads to improved outcomes.*

Causation research optimally involves studying people at a similar, well-defined period in the course of their illness (inception cohort) because differences in the duration of the disease/health condition are otherwise difficult to account for. This is important so as to avoid prevalence-incidence bias where the prognosis for chronic or persistent conditions is different from acute conditions [38]. For example, bias would be introduced if recovery after brain injury were modelled in a case series of patients who had suffered their injury at different times in the past (zero-time bias). That is because these cases would have different trajectories for recovery, and the series would be missing those that recover quickly and those that had died (prevalence-incidence bias). An additional concern is the risk of making erroneous conclusions due to reverse causation, i.e. the outcome was actually present when measuring the prognostic determinant and was the reason why the prognostic determinant was present. However, in the case of chronic conditions with uncertain onsets, it can be challenging to define zero-time. For example, incident back pain commonly occurs during childhood and adolescence, making it difficult to study truly incident adult cases [49]. Various other strategies can and have been employed, including redefining zero time as the onset of an episode of back pain or initial care seeking. However, consumers of such causation research should be aware of the potential effects of case mix on the results of those studies.

### Useful statistics in studies of causation

**Fig. 5:**
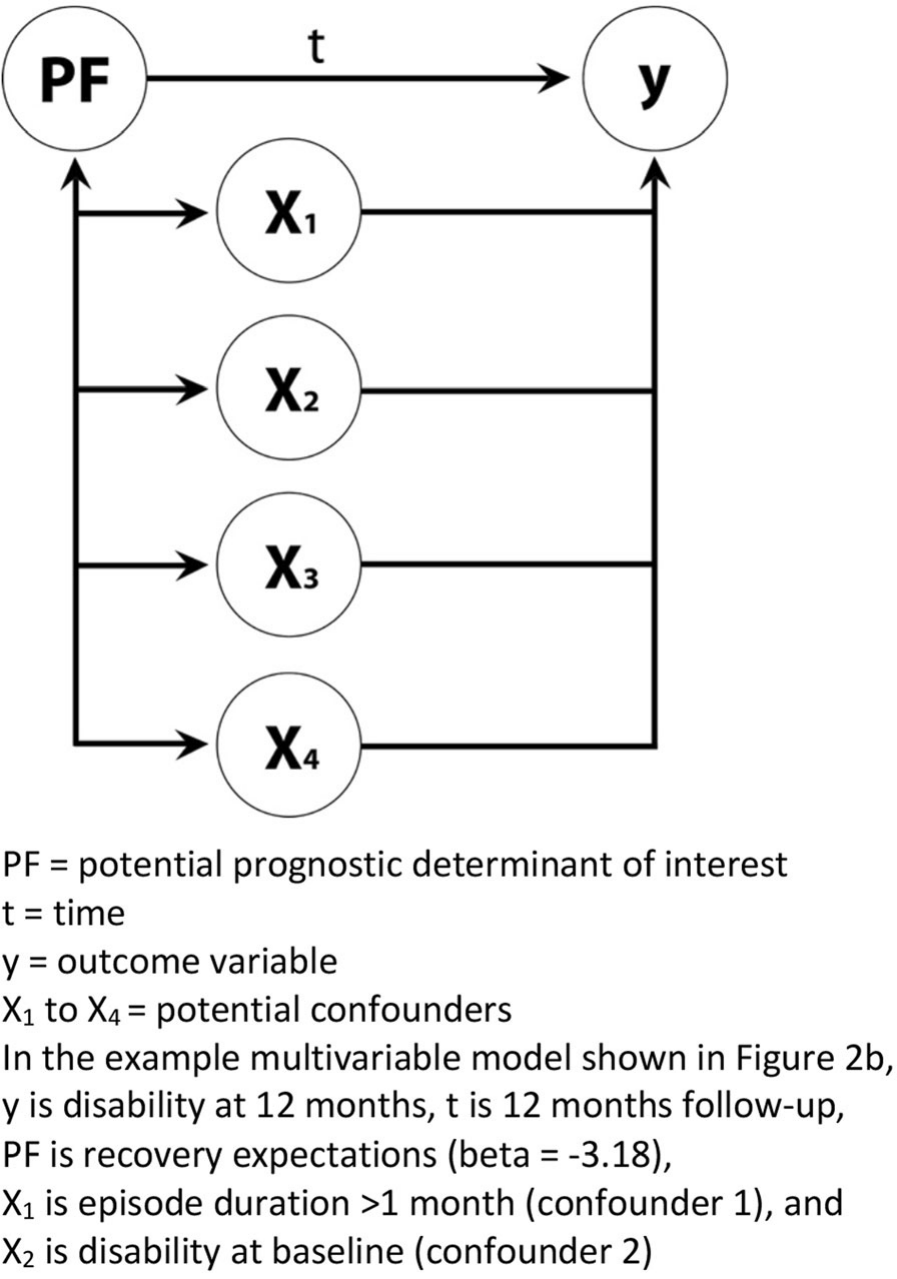
A concept of a multivariable model of a causation study of an independent association

Figure 5 shows a basic conceptual model of one foundational type of causation study, where the relationship between a single prognostic factor and the outcome of interest is tested while controlling for a set of confounding factors (testing an ‘independent association’). Similar to studies of association and prediction, this type of analysis involves a multivariable statistical model, such as that shown in Fig. 2b. What is primarily different is the conceptual understanding of what that model means and how it should be interpreted.

Imagine that the research question for the statistical model in Fig. 2b was to test the hypothesis that baseline recovery expectations (conceptually PF in Fig. 5) has an association with 12-month change scores in functional disability, independent of back pain duration and baseline functional disability (two potential confounders of that association and conceptually X_1_ and X_2_ in Fig. 5). Here the information of interest is only the coefficient, its confidence interval, and the *p*-value for recovery expectations, as the focus is on whether the association between the prognostic factor and outcome remains clinically relevant with a sufficient degree of certainty in the presence of the potential confounders. Estimates for confounders should not be interpreted since there were no pre-specified hypotheses about that relationship. In this example, the beta-coefficient (− 3.18) and its confidence interval (− 2.85 to − 3.50) for baseline recovery expectations indicate that this prognostic factor does have an association with 12-month change scores in functional disability, independent of back pain duration and baseline functional disability, in this sample of people. This statistical model is simplified for applicability across aspects of the prognostic framework, as in a causal context this relationship would be confounded by other factors, potentially including treatment.

The prognostic factor and confounding factors are selected based on prior knowledge and theory. Whereas, prediction research focuses on one optimal model, causation research may test many different models including different models of confounding, mediation or effect moderation that together provide evidence to support a causal relationship. Conceptually, randomised clinical trials study the prognostic determinant ‘treatment’ and eliminate confounding by assigning treatment by randomisation (confounding factors being balanced across the treatment groups as a by-product of the randomisation).

**Fig. 6:**
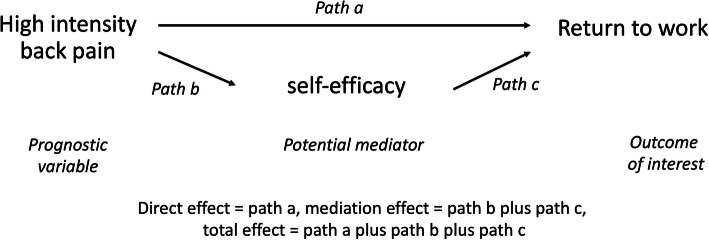
A conceptual model of a causation study of a mediation relationship

Mediation is a formal testing of the hypotheses that a prognostic determinant (such as self-efficacy) acts via an intermediate causal pathway between the exposure or clinical characteristic (such as high pain) and the outcome (such as return to work). In that hypothetical case, part of the reason why high levels of pain hinders return to work, is that high pain has a negative impact on self-efficacy, and low self-efficacy, in turn, hinders return to work (Fig. 6). Mediation analyses are about understanding causal mechanisms and therefore, it is a part of causal research and not part of studies of prediction. Mediation can be part of intervention studies where mechanisms of action are explored within randomised clinical trials. Conceptually, mediators of treatment effect are modifiable prognostic determinants that, when modified by the treatment (Path c in Fig. 6), alter outcome (Path c) [50].

Moderation can be also important in causal studies by modifying the effect of a prognostic factor. The distinction between mediation and moderation in this context being that the score of the mediator is changed by exposure to the prognostic determinant, whereas in moderation the moderator (for example, age) influences the effect of the prognostic factor on the outcome but the moderator’s score is not changed by exposure to the prognostic determinant [51].

We have used the terms ‘exploratory’ (description, association, and prediction model development), ‘confirmatory’ (prediction model external validation’, ‘causal’ and ‘clinical application’) to signal different intentions and different strength of inferences that can be drawn across these phases of research. We have also used examples to illustrate that it is the type of research question that drives the interpretation and that the type of research question determines what parts of the statistical model that are meaningful in that context.

## Conclusions

The aim of this article was to show how different categories of prognostic research are related to and inform each other, and how important it is to understand the differences between association, prediction and causation. Clarity about these aspects can help provide direction about what statistical parameters and interpretations are meaningful in the context of specific research questions. Our intention was to help clarify some of the issues in prognostic research that still are poorly implemented and to stimulate discussion about how conceptual frameworks for prognostic studies can be strengthened to improve the design and interpretation of these types of studies.

## Supplementary information

**Additional file 1.** Overlap between our prognostic research conceptual framework and the phases detailed in the PROGRESS series.

## Data Availability

Data sharing is not applicable to this article as no datasets were generated or analysed during the current study.

## Abbreviations

PROGRESS: Prognosis Research Strategy
TRIPOD: Transparent Reporting of a multivariable prediction model for Individual Prognosis Or Diagnosis statement
CHARMS: CHecklist for critical Appraisal and data extraction for systematic Reviews of prediction Modelling Studies
ROC-curve: Receiver Operating Characteristic curve
Root MSE: Root Mean Squared Error
