# Supplementary material for: A conceptual framework for prognostic research
Source: BMC Med Res Methodol. 2020 Jun 29;20:172. doi: 10.1186/s12874-020-01050-7 (PMC7325141; doi:10.1186/s12874-020-01050-7)

**Additional file 1**

Overlap between our prognostic research conceptual framework and the phases detailed in the PROGRESS series


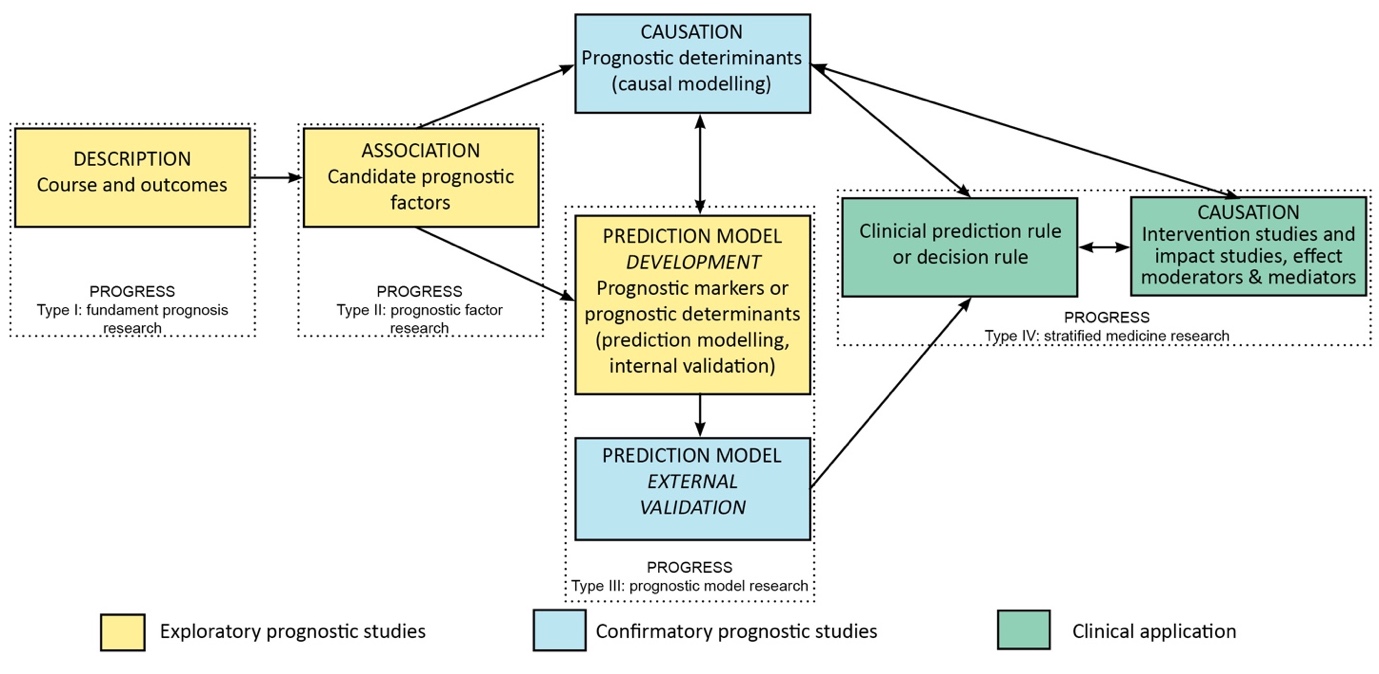

Supplement: Supplementary file 1 — Additional file 1. Overlap between our prognostic research conceptual framework and the phases detailed in the PROGRESS series. [file 12874_2020_1050_MOESM1_ESM.docx]
